# Somatic symptom and related disorders in children and adolescents: evaluation of a naturalistic inpatient multidisciplinary treatment

**DOI:** 10.1186/s13034-018-0239-y

**Published:** 2018-06-28

**Authors:** Pola Heimann, Beate Herpertz-Dahlmann, Jonas Buning, Norbert Wagner, Claudia Stollbrink-Peschgens, Astrid Dempfle, Georg G. von Polier

**Affiliations:** 1 Department of Child and Adolescent Psychiatry, Psychosomatics and Psychotherapy, RWTH Aachen University, Aachen, Germany; 2 Department of Pediatrics, RWTH Aachen University, Aachen, Germany; 3 Department of Medical Informatics and Statistic, University Schleswig-Holstein, Kiel, Germany

**Keywords:** Somatic symptom disorder, Multidisciplinary treatment, School attendance, Pain coping strategies, Comorbidity

## Abstract

**Background:**

This naturalistic study assesses the effectiveness of inpatient multidisciplinary treatment of children and adolescents with somatic symptom disorders (SSD) and investigates the role of pain coping strategies and psychiatric comorbidity (anxiety, depression).

**Methods:**

Sixty children and adolescents (mean age 14.4 years) with SSD who underwent inpatient multidisciplinary treatment were assessed regarding their school attendance, levels of discomfort, coping strategies and psychiatric comorbidity (depression, anxiety) at pretreatment, discharge and 6 months following treatment.

**Results:**

At discharge, the children and adolescents reported improvements in their level of discomfort, psychiatric comorbidities (anxiety, depression) and pain coping strategies, with medium to large effect sizes. Six months following treatment, the improvements remained stable, including significantly higher school attendance rates (*d* = 1.6; *p* < 0.01). Improvement in pain coping was associated with increased school attendance.

**Conclusion:**

Inpatient multidisciplinary treatment is effective in reducing levels of discomfort, psychiatric comorbidity (anxiety, depression), and school absence and in improving coping strategies.

## Background

Somatic symptom disorders (SSD) describe a heterogeneous entity, though the terminology has changed over the years [1, 2]. In the present article they include somatoform disorders, dissociative (or conversion) disorders and somatic disorders with psychiatric comorbidity. Somatic symptom disorders lead to significant functional and emotional impairments e.g., school absence, high socioeconomic costs and frequent use of healthcare services [3, 4]. Recently, increased numbers of children and adolescents suffering from somatic symptom complaints with functional impairments have been reported by van Geelen and colleagues [5]: between 1988 and 2011 the percentage of boys with psychosomatic problems larger than 90th percentile increased from 5.0 to 9.1% and in girls from 16.7 to 24.5% (2619 adolescents included). In particular, factors related to treatment outcomes are poorly understood, and there is a need for further research in this field [3].

In the fifth edition of the Diagnostic and Statistical Manual of Mental Disorders (DSM-5), a new category was introduced known as “somatic symptom and related disorders”. The DSM-5 emphasizes a significant functional impairment, as well as excessive thoughts, feelings and behaviors related to somatic symptoms, while the absence of a medical explanation for the symptoms is no longer necessary [6].

In contrast to the DSM-5, the International Statistical Classification of Diseases and Related Health Problems ICD-10 defines SSD in different categories e.g., somatoform disorder (F45.x) and dissociative and conversion disorder (F44.x) [7].

The most common symptoms reported by children and adolescents with SSD include pain, fatigue, faintness and nausea [8–10]. Specifically, chronic somatic pain (headache, recurrent abdominal and musculoskeletal pain) appears to be very frequent, with up to 25% of children and adolescents being affected in general population samples, including the “German Health Interview and Examination Survey for Children and Adolescents” (KiGGS) [8, 11–13]. Conversion disorders are less frequent, with a prevalence varying between 1–4% and up to 10%, as measured in a pediatric neurological unit [14, 15]. Moreover, data from the KiGGS-survey showed that up to 10.8% of children and adolescents suffer from a chronic somatic disorder and show a threefold increased risk of developing psychiatric comorbidities compared to healthy controls [13]. Likewise, children and adolescents with an SSD show an increased risk of developing psychiatric comorbidity, especially anxious or depressive symptoms [11, 16]. Moreover, adolescents with affective, anxiety and behavior disorders are at risk of developing somatic symptoms such as chronic pain; on the other hand depression and anxiety disorders can be a consequence of chronic pain [17, 18]. Up to 50% of children and adolescents with SSD suffer from psychiatric comorbidity [2]. In addition, affected children and adolescents often face functional long-term impairments resulting in poor academic achievements, an increased risk for later medical treatment and vocational impairment [8, 16, 19]. The emotional burden seems to have an important influence on the long-term treatment outcome [20]. This highlights the importance of consideration and treatment of psychiatric comorbidities during the inpatient multidisciplinary treatment.

Treatment often appears to be unsatisfactory to patients, families and healthcare professionals due to a low acceptance of the concepts of, and interventions for, somatic symptom disorders [1, 8, 11]. For subjects with severe impairments, inpatient multidisciplinary treatment in specialized healthcare units based on a cognitive behavioral approach has been recommended [11, 21]. Notably, a close cooperation among multiple disciplines is warranted, as biological and environmental/social aspects have to be considered [4, 22]. However, a systematic evaluation of treatment approaches is lacking, and specialized healthcare units are rare in Germany and many other developed countries [20]. More specialized multidisciplinary units are needed as unimodal treatment appears to be insufficient for the complexity of the SSD [20]. Compared to sole pediatric and psychiatric treatment, a multidisciplinary treatment approach facilitates a highly specialized treatment and ensures a close collaboration between pediatricians and psychiatrists. Previous studies predominantly focused on chronic somatic pain, while studies investigating SSD, including dissociative (or conversion) disorders and somatic disorders with psychological factors, are scarce [21].

Research of chronic somatic pain in children and adolescents has demonstrated that inpatient multidisciplinary treatment is effective for improving pain intensity, school absence and further pain-related disabilities (e.g., social activities, sports, sleep) [23]. Improvement of pain coping appears to have a strong effect on pain-related treatment outcomes e.g., pain intensity [24–26]. A recently published meta-analysis by Bonvanie and colleagues [21] demonstrated the effectiveness of psychological treatment in improving symptom severity, disability and school attendance at posttreatment and follow-up in children and adolescents with functional somatic symptoms. The type of symptoms did not seem to influence the outcomes [21]. Despite these promising results, research on multidisciplinary treatment of children and adolescents with SSD is scarce, and the mediators of these treatment processes are still not well understood. In addition, an interpretation of the existing studies is limited due to the heterogeneity of the measures used and a lack of data concerning the long-term treatment effects regarding psychosocial functioning (e.g., school attendance) and psychiatric comorbidity [4, 12, 21, 27].

Thus, our study focused on the evaluation of inpatient multidisciplinary treatment of SSD covering all disorders enumerated in DSM-5, with a particular evaluation of distress and impairment (i.e., school absence). In detail, the aims of our study were twofold: first, we aimed to evaluate the effectiveness of an inpatient interdisciplinary treatment for children and adolescents with somatic symptom disorders. The multidisciplinary team consisted of child & adolescent psychiatrists, pediatricians, clinical psychologists, physiotherapists, occupational therapists and nurses. The outcome parameters were a reduction in somatic complaints and psychiatric comorbidity (anxiety, depression) at discharge and upon a 6-month follow-up after treatment completion. At this assessment, school attendance was also evaluated. Second, we aimed to assess the impact of coping strategies and comorbid psychiatric symptoms (depression, anxiety) on changes in functional impairment (i.e., school attendance) and the level of discomfort.

## Methods

Patients aged 8–18 years with somatoform disorders, dissociative disorders or chronic somatic disorders with psychiatric comorbidity who were referred to our somatic symptom unit were eligible for inclusion. The requirements for admission included a complete pediatric diagnostic evaluation and an appointment with a member of the treatment team to discuss the indication of inpatient treatment and treatment goals. The exclusion criteria included insufficient knowledge of the German language, duration of treatment of less than 14 days and severe psychiatric comorbidity, such as acute suicidal ideation or psychosis. Regular treatment attendance at individual and group therapies was a precondition for admission and continued participation in the treatment program. Patient assessments were conducted upon admission (T1), discharge (T2) and 6 months following treatment (T3). The local ethics committee approved the study in accordance with the Declaration of Helsinki.

### Sample

Seventy-three individuals were screened over a 16-month period, and 60 patients were eligible for inclusion. Eight patients cancelled treatment prematurely, and five patients had to be transferred to the child and adolescent psychiatric unit due to severe psychiatric disorders. Forty-five (75%) of all included patients participated in the follow-up assessment. Study completers did not differ from non-completers in terms of age, sex, distribution of disorders or missed school days upon admission (T1). The details are presented in Table 1.

**Table 1:** Demographic and clinical data

	Mean [SD]
Sample size (n)	60
Female (%)	56.7
Age	14.43 [2.0]
Duration of treatment (days)	48.15 [19.7]
*Diagnostic distribution*	*N* [%]
A primary diagnosis	
Somatoform disorder (F45.x)	47 [78.3]
Dissociative (conversion) disorder (F44.x)	4 [6.7]
Other pediatric diagnosis^a^	9 [15]
B comorbidities	
Depressive episode (F32.x)	27 [45]
Phobic/other anxiety disorder (F40.x/F41.x)	24 [40]
Attention deficit hyperactivity disorder (F90.x)	22 [36.7]
Other F^b^—diagnoses	23 [38.3]

^a^ Rheumatic diseases (n = 2), migraine (n = 1), obesity (n = 5), chronic inflammatory bowel syndrome (n = 1)

^b^ Obsessive–compulsive disorder (F42.x, n = 2), reaction to severe stress and adjustment disorder (F43.x, n = 6), specific developmental disorders of scholastic skills (F81.x, n = 4), mixed disorders of conduct and emotions (F92.x, n = 1), emotional disorders with onsets specific to childhood (F93.x, n = 1), tic disorders (F95.x, n = 1), somatoform disorder (F45.x, n = 1)

### Measures

#### School attendance

School attendance was assessed in accordance with a scheme proposed by Hechler et al. (2014) over the 4 weeks prior to admission and over the 4 weeks prior to the 6-month follow-up [37]. School absence was categorized by the amount of missed school days: none (0–1 days), moderate (2–5 days) and high (6–20 days). School attendance was assessed via self-and parental reports.

#### PPCI

The German version of the revised Pediatric Pain Coping Inventory (PPCI-R), a validated self-report questionnaire, was used to assess pain coping strategies in the children and adolescents [25, 28]. The PPCI-R considers the following subscales: cognitive self-instruction, seeking of social support and passive coping strategies. Passive coping strategies and seeking of social support are defined as behavior-related strategies, while positive self-instruction is defined as a cognitive strategy. The summed scores were used for statistical analyses. Lower numbers indicated more adaptive coping strategies [25].

#### Level of discomfort

The German “Giessen physical complaints inventory for children and adolescents” (GBB-SB) is a self-report questionnaire designed to assess subjective somatic complaints [29]. The GBB-SB includes fatigue, gastric and cardiovascular complaints, rheumatic pain, and cold symptoms that altogether provide a total score of the complaints [30]. The subjective perception of somatic complaints often differs from the clinical findings, especially in somatoform disorders. *T* levels, in accordance with the German norms referred to in the manual, were used for statistical analyses.

#### Anxiety

The German version of the Spence Children’s Anxiety Scale (SCAS), a self-report questionnaire, was used to measure overall anxiety and includes the following six subscales: generalized anxiety, panic/agoraphobia, social phobia, separation anxiety, obsessive–compulsive disorder and physical injury fears [31, 32]. The total SCAS sum scores were used for statistical analyses.

#### Depression

The German Children’s Depression Inventory (DIKJ), a self-assessment questionnaire, was used to measure the severity of depressive symptoms [33]. *T* levels, in accordance to the norms reported in the manual, were used for statistical analyses.

#### Treatment

For highly affected children and adolescents with SSD, an interdisciplinary inpatient treatment (Monday until Friday) based on cognitive behavioral treatment strategies was recommended [21, 34]. The primary goal of the treatment was to develop adaptive pain coping strategies and to facilitate a return to everyday adolescent life (e.g., regular school attendance, sports, and social activities). The treatment comprised psychotherapy and complimentary therapies such as physiotherapy and social competence training, as well as parental psychoeducation. One important strategy was the supervised gradual exposition to situations in which somatic symptoms frequently occur with the goal to help patients reappraise associated thoughts and feelings increasing somatic symptoms. The parents were invited to participate in the coaching sessions to learn about the individual pathogenesis of SSD and to understand how to reduce overprotective or perpetuate behavior. At the beginning of the treatment, all patients attended a special school at the hospital, with most returning to their classes at their original schools at the end of the treatment. Pharmacological treatment was recommended as an auxiliary intervention in some cases. Of all the patients, 50% (n = 30) received no medication, and 21.7% (n = 13) were treated with a selective serotonin reuptake inhibitor (SSRI) because of comorbid depression (n = 9) and/or anxiety (n = 6). Of all the patients 21.7% (n = 13) were treated with extended-release methylphenidate, and 1.7% (n = 1) were treated with atomoxetine due to comorbid attention deficit hyperactivity disorder. In 5% (n = 3) of all patients, a combination of SSRIs and extended-release methylphenidate because of comorbid depression (n = 2) or anxiety (n = 1) and attention deficit hyperactivity disorder was administered. All patients were drug-naïve at pretreatment. The treatment included regular team meetings to discuss the individual progress of the patients, as well as regular supervisions. The treatment team had prior experience in the therapy of patients with somatic symptom disorders. Before discharge, caregivers were supported in setting up continued care including psychotherapy and child- and adolescent care including medication in some patients. At discharge, all patients had first appointments set for continued ambulant care.

The interdisciplinary inpatient unit in Aachen provides nine treatment units for highly affected children and adolescents. In Germany, inpatient treatment is more widespread compared to similar health care systems in other Western countries with over 6000 psychiatric inpatient treatment units [35]. However, units for children with SSD are scarce in Germany and mostly cared by only one discipline, either child and adolescent psychiatry or pediatrics. The interdisciplinary model presented here is one of the very few in Germany.

### Statistical analyses

IBM SPSS version 23 was used to perform all statistical analyses (IBM Corp., Armonk, N.Y.). Sample characteristics were summarized using descriptive statistics. Descriptive statistics were employed for the categorical variables and also for the means and standard deviations (SDs) for the continuous variables. Three time-points were assessed: admission, discharge and the follow-up (6 months after discharge). A test of the distribution of normality revealed that the values of the DIKJ, SCAS and the number of missing school days were not normally distributed. Wilcoxon signed-rank tests (DIKJ, SCAS, and missing school days) and paired *T*-tests (GBB; PPCI) were conducted to assess changes from pre- to posttreatment (T1–T2) and from pretreatment to 6 months after discharge (T1–T3). Due to the use of multiple tests of the same dataset, we adjusted the alpha level using the Bonferroni–Holm procedure [36], which specified a *p* value of 0.0033 for a test to be considered significant. The resulting output of the group comparisons was used to calculate the effect sizes (Cohen’s d). Additionally, exploratory post hoc analyses were conducted to investigate the influence of (a) age and sex, or (b) baseline levels of depression or discomfort on the changes in coping strategies, levels of discomfort, depression and anxiety. A repeated measures model with sex as between subject factor and time (T1, T3) as within subject factor was used for (a), while linear regression models were used for (b). Finally, using Pearson correlations, we investigated whether changes in school attendance (T1 to T3) were related to changes (T1 to T3) in pain coping (PPCI), discomfort or comorbidity.

## Results

### Sample

The demographic and clinical characteristics of the study sample are presented in Table 1. The ages ranged from 9 to 17 years, with a mean age of 14 years (*SD* = 2.0). Average school absence during the 4 weeks prior to admission was 11.7 days (*SD* = 7.9), and the mean duration of the inpatient treatment was 48 days (*SD* = 19.7). Patients who received medication had more severe symptoms, e.g., days of school absence than those without medication (p < 0.05).

### Treatment effects at discharge

Measures at admission (T1), discharge (T2) and 6 months following treatment (T3) are given in Table 2. Compared with pretreatment, the effects of the treatment at discharge were noted regarding reduced levels of comorbidity with small to medium effect sizes (anxiety: *d *= 0.4; depression: *d *= 0.6). The level of discomfort was reduced with a large effect size (*d *= 0.8).

**Table 2:** Discomfort, pain coping and comorbidity

Measures	Admission	Discharge	Six months after discharge
DIKJ^a^	55.67 [10.08]	49.22 [11.42]*	45.05 [13.73]**
SCAS^b^	26.44 [15.07]	20.08 [17.47]*	15.38 [15.47]**
GBB-SB^b^	59.15 [11.03]	48.96 [14.38]**	48.38 [13.63]**
PPCI^b^	22.86 [8.67]	18.68 [7.14]*	14.21 [7.87]**
PAS^c^	10.20 [4.42]	6.42 [3.78]**	6.11 [3.80]**
SS^c^	4.76 [3.97]	3.33 [2.61]*	1.86 [2.40]**
POS^c^	8.47 [3.29]	9.16 [3.70]	6.31 [4.19]
School absence^d^ [in days]	11.92 [7.05]		2.41 [4.28]**
None	5 (10.2%)		29 (64.4%)
Moderate	6 (12.2%)		10 (22.2%)
High	38 (77.6%)		6 (13.3%)

Means and standard-deviations in brackets

* Significant at *p*_*α*_ < 0.0033 compared with pretreatment data; ** *p* < 0.001

^a^ T-value

^b^ Raw-value

^c^ *PAS* passive strategies, *ss* seeking for social support, *POS* positive self-instruction

^d^ School absence in days for the prior 4 weeks; none = 0–1 days, moderate = 2–5 days, high = > 5 days (%); these subgroups were not included in the comparison tests

### Treatment effects 6 months post treatment

**Fig. 1:**
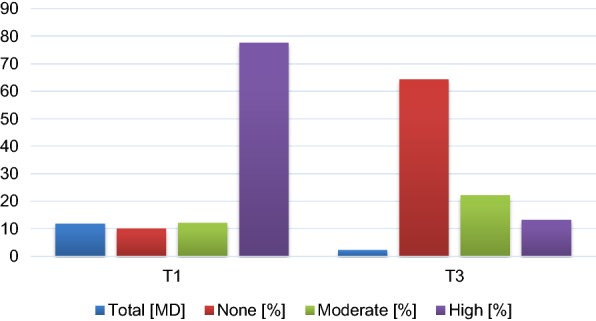
School absence at 4 weeks prior to treatment (T1) and 6 months following treatment (T3). None: 0–1, moderate: 2–5, high: 6–20 school days missed over 4 weeks; (%): percent of all patients; total [MD]: number of missed school days (mean duration over 4 weeks)

Compared with the pretreatment status (admission), treatment effects remained stable 6 months post treatment indicated by significantly reduced levels of discomfort, depression and anxiety and improved coping strategies (Table 2). Moreover, school attendance improved with large effect sizes (*d* = 1.6; Table 2, Fig. 1). Notably, 20 (52%) of the subjects in the high school absence group (> 5 days/4 weeks) were rated as having no school absences (0–1 days/4 weeks) at 6 months posttreatment.

When treatment effects 6 months post treatment were compared with the status upon discharge, within subjects’ comparisons indicate no changes in depression (*t* = 1.16; *p* = 0.26), anxiety (*t* = 0.96; *p* = 0.34) and levels of discomfort (*t* = − 0.51; *p* = 0.61) and further improved coping strategies (PPCI; *t* = 3.46; *p* = 0.001), mainly attributable to reductions in self instructions (POS; *t* = 3.47; *p* = 0.001).

Exploratory post hoc analyses regarding correlates of treatment outcome (i.e., level of discomfort 6 months posttreatment and school attendance), age and gender indicated the following:

- Age was not associated with treatment outcome. Boys and girls showed comparable levels of discomfort at baseline (GBB raw sum score; boys mean 61.1, sd 11.3; girls mean 58.5, sd 11.1), however, boys showed larger reductions in levels of discomfort at T3 (boys mean 47.8, sd 13.5; girls: mean 49.6, sd 15.3). This was indicated by a significant repeated measures model with GBB T1/T3 as dependent variable and gender as between subjects factor: GBB was reduced over time (*p *< 0.001), and significantly different between sexes (*p *= 0.02 for the time by sex interaction, overall sex effect *p *= 0.7).
- In a linear regression model, lower levels of discomfort 6 months posttreatment (T3, self-assessment) were predicted by lower depression scores upon admission (T1; *β *= 0.36; *t *= 2.3; *p *= 0.03) but not levels of discomfort upon admission (*p *= 0.14). Since depression scores and levels of discomfort were correlated at T1 (*r *= 0.28; *p *= 0.04), a second model only including depression scores at T1 was calculated and results were largely similar (*β *= 0.41; *t *= 2.7; *p *= 0.01).
- Improved school attendance (i.e., delta school absence: T1–T3) was associated with improved pain coping (T1–T3; *r *= 0.38, *p* = 0.037) but not with decreased levels of discomfort or with depressive or anxious symptoms scores.

## Discussion

The key finding of this study was that, in children and adolescents with SSD, an inpatient interdisciplinary treatment program is highly effective in reducing somatic complaints, increasing school attendance, developing adaptive coping strategies and improving psychiatric comorbidity (depression, anxiety). Furthermore, our findings highlighted the importance of developing adaptive coping strategies that are associated with improved school attendance.

Our findings were consistent with those of previous studies, which demonstrated that children who were highly affected by chronic pain improved in response to inpatient treatment in regard to functional impairment, pain intensity and quality of life [4, 11, 37, 38]. Although there exists strong evidence for the short-term effectiveness of inpatient treatment, research concerning the long-term outcome is rare, and tools for measuring treatment outcomes need to be standardized for better comparability [38]. A meta-analysis by Hechler and colleagues demonstrated that intensive interdisciplinary pain treatment has positive treatment effects on pain intensity, disability and depressive symptoms, though school functioning and anxiety were excluded due to the heterogeneity in the treatment outcome measures [4]. A recent meta-analysis [21] demonstrated the effectiveness of psychological treatment on functional somatic symptoms. Notably, the *type of symptom* was not associated with the effectiveness of treatment. This may be interpreted in support of the findings of this study that included all somatic symptom disorders and pediatric disorders being complicated by psychological factors.

Second, our results support previous findings that inpatient interdisciplinary treatment can substantially reduce school absence [23]. In accordance with prior research showing that 80–90% of patients with chronic pain show a significant improvement in school attendance, our findings suggest similar results, with only 13.3% maintaining high levels of school absence at 6 months after discharge (i.e., 6–20 school days missed over 4 weeks) [23, 39]. As school attendance is highly important for the development of children and adolescents, factors explaining the persistent school absence rates must urgently be identified [23].

Moreover, previous studies have suggested that adaptive coping strategies play an important role in the treatment of recurrent and chronic pain [40, 41]. While associations of pain coping and improvement in affective symptoms have been inconsistent [42, 43], improvements in functional disability and quality of life have been previously reported [43]. Further, our findings regarding the changes in coping are consistent with former research conducted by Hechler and colleagues [41], who reported identical findings, i.e., reduced passive pain coping and seeking of social support after a multimodal inpatient treatment, with positive self-instruction being maintained. Thus, associations of improved behavioral coping strategies (more active coping, less seeking of social support) with improved school attendance implies that one promising treatment approach could involve focusing on helping patients become more active in reducing passive coping. One element of our multidisciplinary treatment that helped patients become active were the several treatments of physiotherapy per week, in both group and individual settings. Moreover, this treatment fostered positive social experiences and skills within the patient group, both due to the inpatient setting and specifically in social competence trainings and in supervised group activities. Future research should further investigate which specific treatment strategies are most beneficial in improving behavioral coping. While the importance of coping has been demonstrated in adolescents with chronic somatic pain, our results suggested that improvements in coping strategies are an important treatment target in children and adolescents with SSD as well.

Concerning associations of gender and pain coping, one study demonstrated that reduced seeking of social support was associated with reduced pain intensity in girls but not in boys [41]. In our study, boys showed larger improvements in pain coping strategies compared to girls; however, due to the sample size, we did not explore gender-specific associations of pain coping and the level of discomfort. More research is needed to investigate the role of gender in pain coping, as gender-specific coping strategies may improve the treatment outcomes [32].

Lastly, concerning comorbid affective disorders, our findings support previous data showing that over the course of the inpatient treatment, anxiety and depression improved significantly [4, 11, 37]. However, previous research has been heterogeneous and has not always included specific psychiatric treatments of comorbidities, as conducted in this study. In some cases, treatment of psychiatric comorbidity involved the use of medication that was not previously administered to the patients. This must be considered when interpreting the results of our study. Of note, the improvements in psychiatric comorbidity were not significantly related to changes in school attendance or the level of discomfort. While this finding has been reported previously [44], it should be interpreted with caution due to the sample size this non-finding is based on.

The strengths of this study include the use of the standardized assessment of treatment effects and the inclusion of a 6-month follow up. The limitations apply to the study design, as it evaluates a “natural” clinical treatment program lacking randomization, a control group, a standardized therapeutic manual and structured measures of treatment adherence. Future studies with larger sample sizes are needed to assess which specific treatment strategies add to the effectiveness of an inpatient interdisciplinary treatment and also to investigate the role of coping strategies in more detail.

## Conclusion

Inpatient interdisciplinary treatment combining pediatric and psychiatric knowledge and skills is highly effective for SSD in children and adolescents regarding the reduction in somatic complaints, emotional distress, and school absence and the improvement of coping strategies. Untreated somatic symptom and related disorders often result in long-term impairment in affected children and adolescents, including poor academic achievements, an increased risk for later medical treatment and vocational impairments. Thus, it is mandatory to overcome potential barriers to treatment in order to take advantage of multidisciplinary treatment approaches. In this regard, the education of health professionals, teachers and parents regarding recognition and treatment opportunities of SSD is necessary to avoid unnecessary and costly physical examinations, to shorten the duration of illness and to facilitate access to professional treatment.

## Abbreviations

SSD: somatic symptom and related disorders
DSM-5: fifth edition of the Diagnostic and Statistical Manual of Mental Disorders
ICD-10: tenth edition International Statistical Classification of Diseases and Related Health Problems
ICD-11: eleventh edition International Statistical Classification of Diseases and Related Health Problems
KiGGS: German Health Interview and Examination Survey for Children and Adolescents
PPCI-R: Revised Pediatric Pain Coping Inventory
GBB-SB: Giessen physical complaints inventory for children and adolescents
SCAS: Spence Children’s Anxiety Scale
DIKJ: Children’s Depression Inventory
SSRI: selective serotonin reuptake inhibitor
SDs: standard deviations
PAS: passive strategies
SS: seeking for social support
POS: positive self-instruction
MD: mean raw sum difference
