# Correction to: Somatic symptom and related disorders in children and adolescents: evaluation of a naturalistic inpatient multidisciplinary treatment

**DOI:** 10.1186/s13034-018-0253-0

**Published:** 2018-11-01

**Authors:** Pola Heimann, Beate Herpertz-Dahlmann, Jonas Buning, Norbert Wagner, Claudia Stollbrink-Peschgens, Astrid Dempfle, Georg G. von Polier

**Affiliations:** 1 Department of Child and Adolescent Psychiatry, Psychosomatics and Psychotherapy, RWTH Aachen University, Aachen, Germany; 2 Department of Pediatrics, RWTH Aachen University, Aachen, Germany; 3 Department of Medical Informatics and Statistic, University Schleswig-Holstein, Kiel, Germany

## Correction to: Child Adolesc Psychiatry Ment Health (2018) 12:34 10.1186/s13034-018-0239-y

Following publication of the original article [1], the authors flagged an error in the “Authors’ contributions” section of the article.

The (incorrect) text in this section says:

- BH and JB conceptualised the study. JB supervised data collection, PH, AD and GGP analysed and interpreted the data and drafted the manuscript. BH, NW, and CS revised the manuscript critically. All authors read and approved the final manuscript.

As such, please be advised that the correct text (*correction in bold) is:

- BH and JB conceptualised the study. JB supervised data collection, PH, AD and GGP analysed and interpreted the data and **PH drafted the manuscript**. BH, NW, and CS revised the manuscript critically. All authors read and approved the final manuscript.
